# Multifrequency Magnetic Resonance Elastography of the Abdominal Aortic Wall: Segmental, Positional and Flow Effects

**DOI:** 10.1002/nbm.70401

**Published:** 2026-09-17

**Authors:** Yanglei Wu, Yikun Wang, Jakob Schattenfroh, Tom Meyer, Thomas Elgeti, Yining Wang, Jens Vogel‐Claussen, Xuan Wang, Ingolf Sack, Jing Guo

**Affiliations:** ^1^ Department of Radiology Charité ‐ Universitätsmedizin Berlin Germany; ^2^ Department of Radiology, State Key Laboratory of Complex Severe and Rare Diseases Peking Union Medical College Hospital, Chinese Academy of Medical Sciences and Peking Union Medical College Beijing China

**Keywords:** abdominal aorta, aortic wall stiffness, examination position, hemodynamics, magnetic resonance elastography, shear wave speed

## Abstract

Current aortic magnetic‐resonance‐elastography (MRE) infers wall stiffness indirectly from waveguide effects. This study aimed to evaluate the feasibility of abdominal aortic MRE for direct assessment of wall stiffness and its dependence on segmental region, patient position, and hemodynamic parameters. This prospective study examined 11 healthy young male volunteers using MRE to evaluate two anatomical segments of the abdominal aorta: the supraceliac (SC) and infrarenal (IR) segments. The volunteers underwent examinations in both the supine and prone positions. An ECG trigger delay was implemented for MRE to minimize the blood signal in the lumen. Three‐dimensional wavefields were acquired at three mechanical frequencies (70, 80, and 90 Hz), and shear wave speed (SWS) maps were reconstructed as a stiffness surrogate. In addition, blood flow‐velocity MRI was performed assess hemodynamic parameters. Test–retest examinations were performed in a subgroup to evaluate repeatability of aortic MRE. Aortic wall MRE was successful in all participants with good repeatability (ICC = 0.76). Mean wall SWS was higher in SC than IR segments at supine (1.47 ± 0.07 vs. 1.39 ± 0.13 m/s, *p* = 0.035) and prone positions (1.40 ± 0.09 vs. 1.33 ± 0.10 m/s, *p* = 0.046) with higher values in supine SC segments (*p* = 0.019). Flow measurements revealed higher peak velocity and peak flow in SC than IR segments (all *p* < 0.001) with significant effects of examination position on peak velocity and cross‐sectional area in SC (all *p* < 0.05). In prone position, peak velocity correlated negatively with aorta stiffness (SC: r = −0.75, *p* = 0.008; IR: r = −0.69, *p* = 0.019). This study demonstrated abdominal aortic MRE for directly depicting wall stiffness with minimal blood contribution. Segment‐specific aortic wall stiffness was sensitive to circulatory loading and vascular distensibility. In a clinical context, high‐resolution multifrequency MRE could serve as an imaging marker for vascular health and abdominal aortic risk stratification.

## Graphical abstract

In vivo abdominal aortic wall–specific MRE enables reproducible wall stiffness mapping while minimizing the intraluminal signal interference, revealing segment‐ and posture‐dependent stiffness patterns that complement morphology and flow measures for clinical evaluation of abdominal aortic wall health.

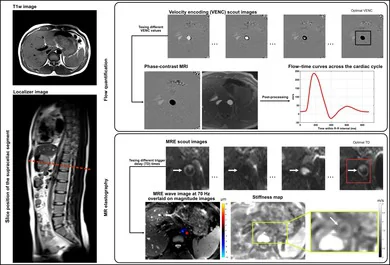

## 1 Introduction

Arterial stiffness is a hallmark of vascular aging and an independent predictor of cardiovascular events [1, 2]. An increase in stiffness of the abdominal aorta has been observed during the development of abdominal aortic aneurysms (AAA) [3]. Conversely, a reduction in aortic wall stiffness in established aneurysms has been associated with disease progression and adverse events [3, 4]. These findings show the close connection between aortic wall stiffness and extracellular matrix remodeling.

Carotid–femoral pulse wave velocity (cfPWV) is currently the reference standard for noninvasive arterial stiffness assessment. However, it is an indirect measure of wall biomechanic properties due to its dependence on geometric assumptions and hemodynamic conditions [1, 5]. Catheter‐based pressure measurement provides direct mechanical information but is limited in routine applications due to its invasive nature [6]. Imaging surrogates derived from CT or MRI, such as distensibility or strain indices, provide complementary data. However, these parameters primarily reflect luminal changes rather than intrinsic wall properties [7, 8].

Elastography has emerged as a noninvasive imaging modality that can quantitatively map aortic stiffness in vivo [4, 9, 10, 11, 12, 13, 14, 15, 16, 17, 18, 19, 20, 21]. In most of the studies using ultrasound elastography [9, 10, 13] or magnetic resonance elastography (MRE), technical challenges arise due to the thin walls in the presence of pulsatile flow. Therefore, elastography traditionally assesses biomechanical parameters of the aortic wall based on waveguide effects encoded within the intraluminal blood. This approach, however, does not fully capture wall‐intrinsic stiffness as it relies on the geometrical boundary conditions of the waveguide [4, 11, 12, 14, 15, 16, 17, 18, 19, 20, 21]. From a technical perspective, the biomechanical properties quantified by MRE could reflect microstructural changes on a submillimeter scale [22], thereby enabling investigation of the abdominal aortic wall. Furthermore, the potential variability in abdominal aorta stiffness across anatomical segments, which may indicate disease progression—such as aneurysm development, sac expansion, or increased rupture or repair risk [4, 23]—remains incompletely understood, particularly with respect to wall stiffness rather than lumen‐based indices. Additionally, the impact of examination position on aortic stiffness, which is relevant in clinical settings such as prone ventilation or surgical positioning, remains unexplored.

This present prospective study aimed to investigate the feasibility and clinical potential of MRE for assessing the stiffness of the abdominal aortic wall at different anatomical segments and under different examination positions. The objectives of this study were threefold: (1) to evaluate the feasibility of abdominal aortic wall MRE in healthy volunteers; (2) to compare the stiffness of the abdominal aortic wall between distinct anatomical segments and to assess the influence of supine versus prone examination position on the abdominal aortic wall stiffness; and (3) to explore the relationship between wall stiffness and hemodynamic parameters obtained from flow‐quantification MRI.

## 2 Materials and Methods

This prospective study was approved by the institutional review board and local ethics committee (Application ID: EA2/155/24), and written informed consent was obtained from all participants. All procedures were performed in accordance with the Declaration of Helsinki.

### 2.1 Participants

Eleven healthy adult male volunteers with no history of cardiovascular disease were included in this study. Exclusion criteria were cardiovascular risk factors (hypertension, diabetes mellitus, hyperlipidemia, active smoking, and body mass index [BMI] > 30 kg/m^2^) and contraindications to MRI. Baseline characteristics including age, height, weight, systolic blood pressure (SBP), diastolic blood pressure (DBP), and heart rate (HR) were recorded for each subject before imaging. BMI was calculated as weight divided by height squared. Mean arterial pressure (MAP) was derived as DBP + (SBP − DBP) / 3, and pulse pressure (PP) as SBP − DBP.

### 2.2 Image Acquisition and Postprocessing

**FIGURE 1:**
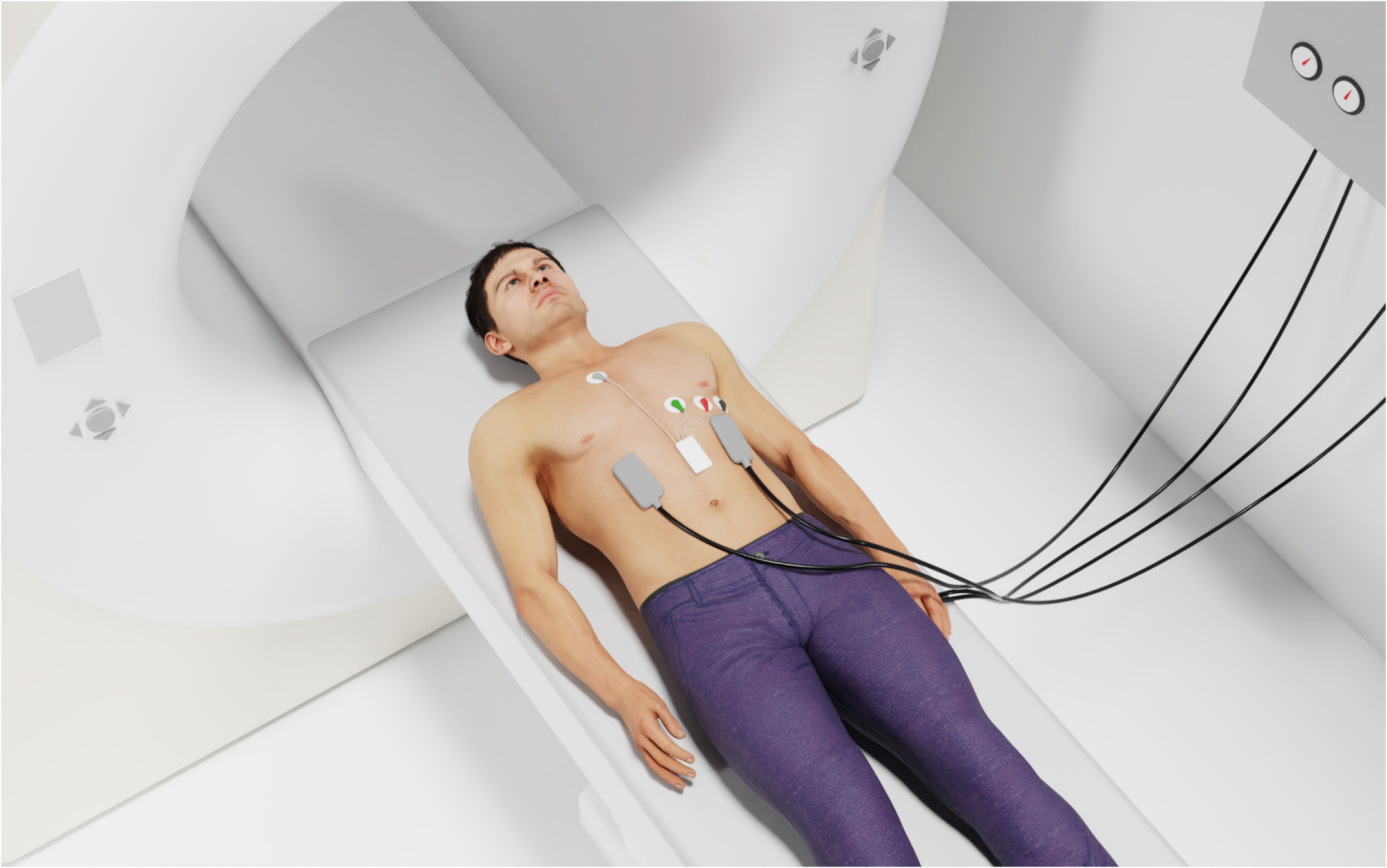
Setup of aortic wall MRE with four pressurized‐air drivers placed in apical and posterior positions onto the thorax.

**FIGURE 2:**
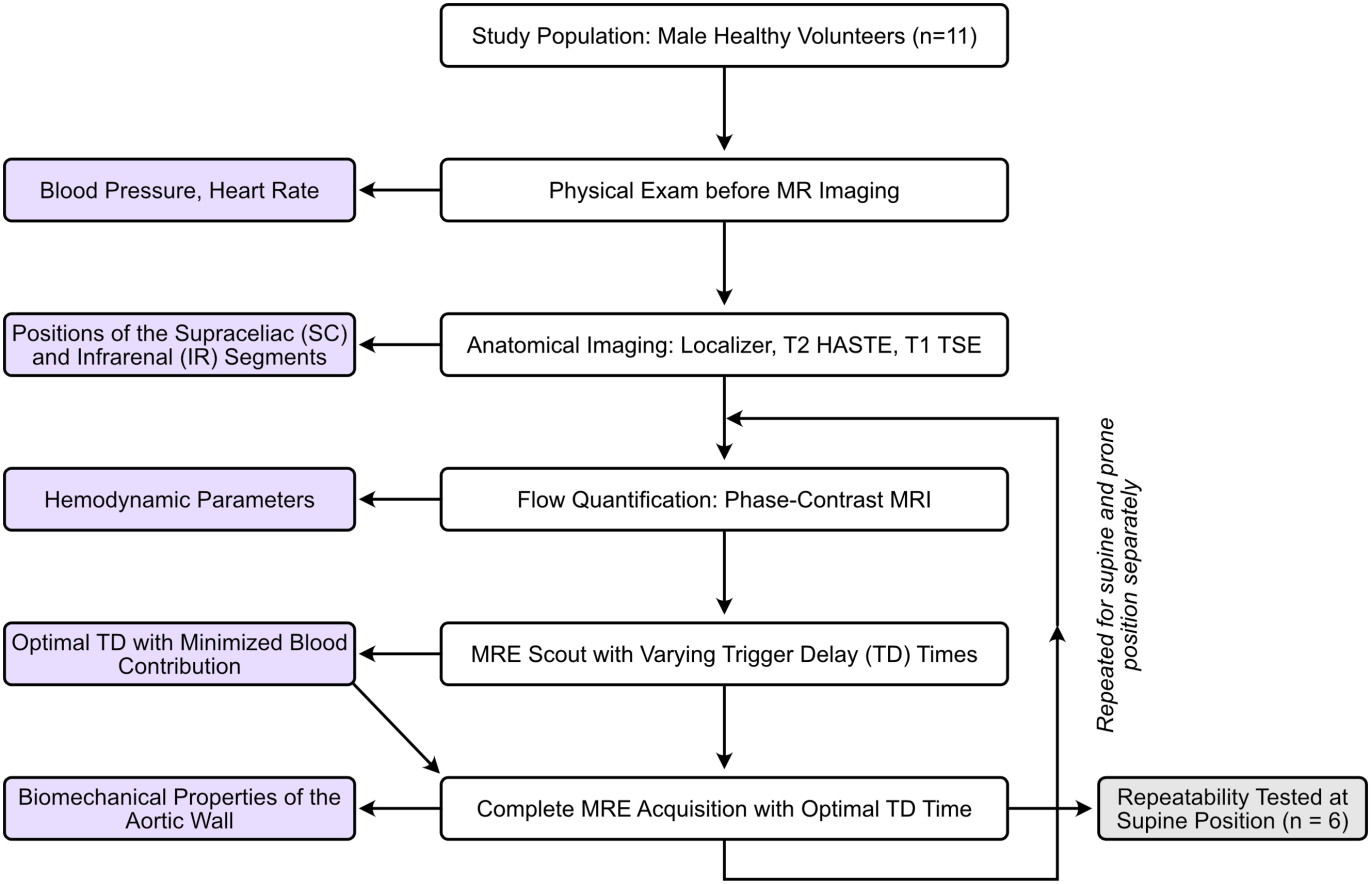
Study flowchart.

All images were acquired on a 3.0‐T clinical MRI scanner (MAGNETOM Lumina, Siemens Healthineers, Erlangen, Germany) equipped with a 24‐channel spine coil and a 12‐channel phased‐array surface coil. For MRE, mechanical vibrations were generated and transferred with four pressurized‐air powered drivers placed around the abdomen (Figure 1), similarly to the placement described in a previous study [24]. The whole imaging protocol was repeated with the participants lying in both supine and prone positions. For each position, two abdominal aortic segments were investigated: the supraceliac segment (SC, above the celiac trunk) and the infrarenal segment (IR, below the origin of the renal arteries). These locations were selected due to their susceptibility to aneurysms and plaque [25]. The axial image slices used for anatomy, flow‐quantification and MRE were planned on sagittal localizer images, oriented perpendicular to the longitudinal axis of the abdominal aorta. The precise slice positions were subsequently refined using axial localizer images to align with the predefined SC and IR segments. The imaging flowchart is shown in Figure 2 and the imaging protocol included the following sections.

#### 2.2.1 Anatomical Imaging

Anatomical images were acquired under free‐breathing using a three‐plane localizer sequence followed by a transverse T2‐weighted fast half‐Fourier turbo spin‐echo (HASTE) sequence (echo time [TE], 95 ms; repetition time [TR], 1400 ms) for the positioning of the two abdominal aortic segments. Additionally, axial T1‐weighted images were acquired with turbo spin‐echo (TSE) sequence (TE, 27 ms; TR, 700 ms) under breath‐hold.

#### 2.2.2 Flow Quantification

**FIGURE 3:**
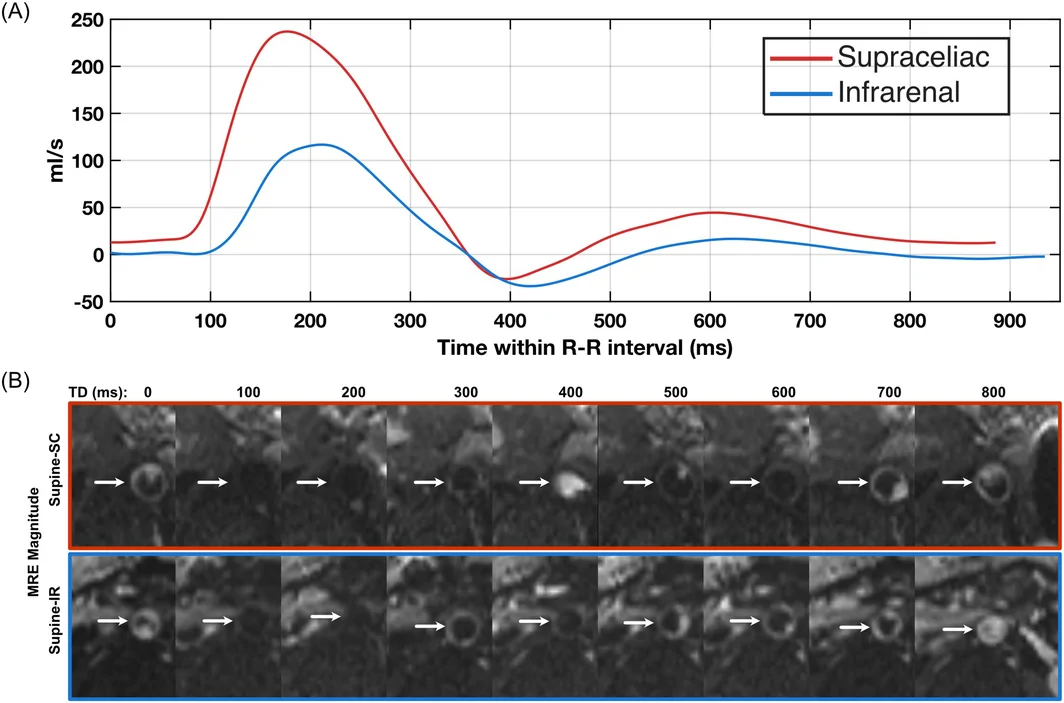
(A) Representative flow–time curves across the cardiac cycle obtained from the flow‐quantification sequence of the supraceliac (SC, in red) and infrarenal (IR, in blue) segments. (B) Series of MRE scout magnitude images of the abdominal aorta (white arrow) in the SC (red) and IR (blue) segments corresponding to different cardiac trigger delay (TD) times, obtained at supine examination position.

Hemodynamic assessment was performed using phase‐contrast MRI with ECG‐synchronized cine acquisition over multiple cardiac cycles under breath‐hold. The velocity encoding (VENC) value for the sequence was determined from a scout scan (ranging from 80 to 140 cm/s in 10 cm/s increments). A single‐VENC, one‐directional (through‐plane) flow encoding scheme was used, yielding flow–time curves across the cardiac cycle (Figure 3A). Post‐processing was conducted with syngo.via MR Cardiac Analysis software (Siemens Healthineers, Erlangen, Germany) to derive the following parameters: peak velocity (cm/s), time‐to‐peak velocity (ms), peak flow (mL/s), and aortic cross‐sectional area (cm^2^). CSA was defined as the cross‐sectional area of the aortic lumen on Flow VENC images. Three CSA metrics were obtained: maximal CSA, minimal CSA and mean CSA across the cardiac cycle.

#### 2.2.3 MRE Acquisition and Processing

To minimize the contribution of the blood and highlight the signal of the aortic wall, an ECG trigger delay (TD) was implemented for MRE acquisition. To find the optimal TD where the blood signal was nulled, a series of MRE scout images with varying TD from 0 to 800 ms in 100‐ms increments were acquired within 25 s under free‐breathing at one time step and one motion encoding direction. The magnitude images of these scout series were visually inspected to identify the trigger delay (TD) that yielded the highest wall signal and the darkest lumen appearance. The corresponding TD value was then selected for the final MRE acquisition (Figure 3B). The TD time was optimized separately for the SC and IR segments.

MRE data were acquired in the axial plane using a free‐breathing spin‐echo‐based echo planar imaging sequence, with one slice per abdominal aortic segment. Wave propagation was encoded at eight vibration phase offsets using a second‐order moment nulling motion encoding gradient (MEG) which was consecutively deployed along all three orthogonal axes of the patient coordinate system. The encoding efficiencies for the driving frequencies of 70, 80, and 90 Hz were 46.97, 57.23, and 65.25 rad/mm, respectively. Further imaging parameters were: TE, 50 ms; TR, 152 ms; voxel size, 2 × 2 × 2 mm^3^, in‐plane acceleration factor 2, and phase partial Fourier 7/8. Data acquisition was synchronized to the cardiac cycle using ECG gating. Respiratory motion was controlled using a navigator‐triggered prospective acquisition correction (PACE) technique with an acceptance window of ±2.0 mm. The mean acquisition time for a single aortic wall segment was approximately four to 5 min, with individual variations resulting from differing R–R intervals and respiratory frequencies. To assess the repeatability of the method, test–retest examinations were consecutively performed in a subgroup of six volunteers at both SC and IR segments in the supine position.

MRE data were reconstructed using the multifrequency inversion method k‐MDEV [26] available at bioqic‐apps.charite.de [27], yielding maps of shear wave speed (SWS, m/s), surrogating tissue stiffness. ROIs were manually drawn using Horos (version 3.3.6; Horos Project, Annapolis, Maryland, USA; available at horosproject.org) by placing an annular region of interest encompassing the aortic wall.

### 2.3 Statistical Analysis

All analyses were performed using R software (version 4.5.1; R Foundation for Statistical Computing, Vienna, Austria). Data distribution was tested with the Shapiro–Wilk test. Normally distributed variables are presented as mean ± standard deviation (SD), and non‐normally distributed variables as median (interquartile range, IQR).

Correlations between SWS and clinical/hemodynamic parameters were first examined using Pearson's or Spearman's method, as appropriate. To account for repeated measures (two aortic segments × two examination positions per subject), repeated‐measures correlation (rmcorr) and linear mixed‐effects models (LME) were applied. In LME, SWS served as the dependent variable, each clinical parameter (age, height, weight, body mass index, systolic and diastolic blood pressure, mean arterial pressure, pulse pressure, and heart rate) was entered separately as a fixed effect, with examination positions and aortic segments (including their interaction) as covariates, and subject‐specific random intercepts to account for within‐subject correlation. In exploratory analyses, condition‐specific linear regressions (supine‐SC, supine‐IR, prone‐SC, prone‐IR) were performed, and *p* values were adjusted for multiple testing using the false discovery rate (FDR, Benjamini–Hochberg). Intraindividual reproducibility was assessed by calculating intraclass correlation coefficients (ICCs). A two‐tailed *p* < 0.05 was considered statistically significant.

## 3 Results

### 3.1 Participant Characteristics

Eleven healthy male volunteers [median age, interquartile range (IQR), 30 (27–30) years; mean BMI, 23.0 ± 2.4 kg/m^2^] were included. Aortic wall thickness estimated from the magnitude images of the phase‐contrast flow sequence during mid‐to‐late diastole was 1.8 ± 0.2 mm (mean ± standard deviation) across all healthy volunteers, with a range of 1.4–2.4 mm. Demographic and physiological parameters are summarized in Table 1.

**TABLE 1:** Summary of participant characteristics (*n* = 11).

Characteristic	Value
Age, years	29 (27–30)
Height, cm	176.6 ± 8.2
Weight, kg	71.7 ± 7.6
Body mass index (kg/m^2^)	23.0 ± 2.4
Systolic blood pressure (mmHg)	127.7 ± 7.8
Diastolic blood pressure (mmHg)	81.3 ± 6.6
Mean arterial pressure (mmHg)	96.8 ± 5.8
Pulse pressure (mmHg)	46.5 ± 8.2
Heart rate (bpm)	65 (60–70)

*Note:* bpm = beats per minute. Variables with normal distribution are presented as mean ± SD, and non‐normally distributed variables are presented as median (IQR).

### 3.2 Quantification of Biomechanical and Hemodynamic Parameters

#### 3.2.1 Regional Biomechanical Properties of the Abdominal Aortic Wall at Different Examination Positions

**FIGURE 4:**
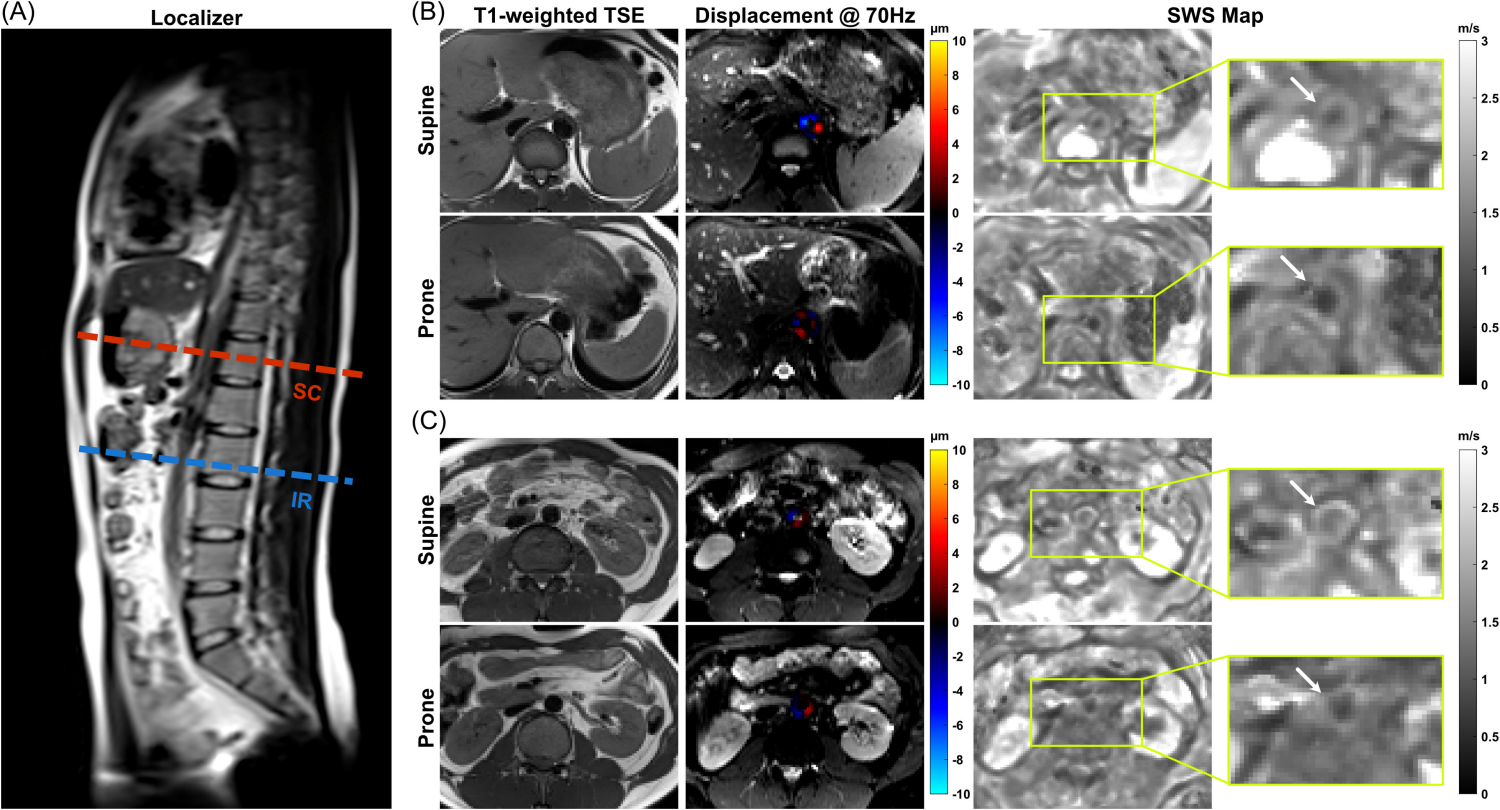
Representative MRI anatomical images as well as MRE magnitude, displacement field, and shear wave speed (SWS) maps of the abdominal aorta are shown in supraceliac (SC) and infrarenal (IR) segments. (A) Sagittal T1‐weighted localizer showing the placement of the two aortic segments (dashed lines): SC (red) above the celiac trunk and IR (blue) below the renal arteries. T1‐weighted turbo spin‐echo (TSE) image, MRE displacement along z‐axis (slice‐select encoding direction) at 70 Hz overlaid on the magnitude images, and MRE SWS map shown in SC (B) and IR (C) segments for supine and prone examination positions.

Representative MRE magnitude images, wave images and the corresponding shear wave speed (SWS) maps at both supraceliac (SC) and infrarenal (IR) segments in both supine and prone positions are shown in Figure 4. The complete displacement field and animated shear‐wave propagation of the same volunteer are shown in Supplemental Figures SF1 and SF2, respectively. The presence of waves is clearly discernible in the aortic wall, and the lumen appears dark in the magnitude images, indicative of effective blood signal suppression. The displacement amplitude within the aortic wall ROI was 10.8 ± 3.0 μm across all subjects and measurements, substantially exceeding the 4 μm threshold for adequate wave propagation proposed by Schattenfroh et al. [24]. The trigger delay times for this volunteer were 800 ms and 700 ms, which corresponded to the period of dynamic flow curves with low flow velocity and minimal vessel wall movement (Figure 3B).

**FIGURE 5:**
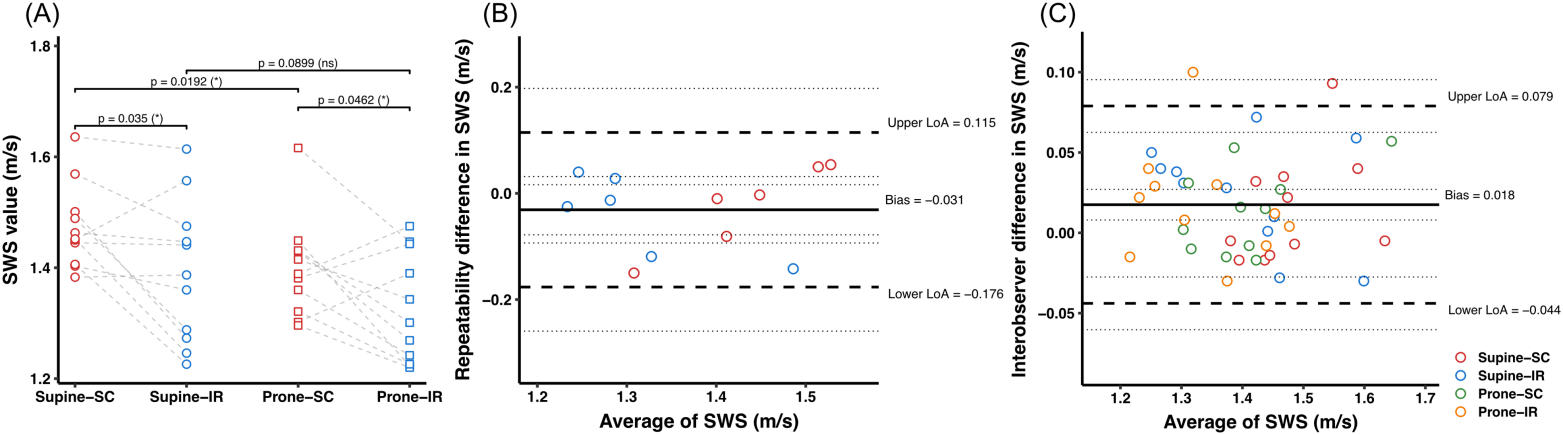
(A) Group comparison of the shear wave speed (SWS) values measured at the supraceliac (SC, in red) and infrarenal (IR, in blue) segments in both supine (open circle) and prone positions (open square). Colored dots represent individual volunteers, and gray dashed lines connect paired measurements within the same subject. (B) Bland–Altman plot showing intraindividual repeatability of MRE measurements. The solid line represents the mean bias (−0.031 m/s), and dashed lines indicate the 95% limits of agreement (LoA: −0.176–0.115 m/s). (C) Bland–Altman plot showing interobserver reproducibility of SWS measurements derived from independent aortic wall segmentations. The solid line represents the mean bias (0.018 m/s), and dashed lines indicate the 95% limits of agreement (LoA: −0.044–0.079 m/s). Data points represent measurements from different anatomical segments and body positions.

In Figure 5A, group analysis showed that mean SWS values were significantly higher at SC segment compared with the IR segment in the supine position (1.47 ± 0.07 m/s vs. 1.39 ± 0.13 m/s, *p* = 0.035) and in the prone position (1.40 ± 0.09 m/s vs. 1.33 ± 0.10 m/s, *p* = 0.046). Furthermore, SWS was higher in the supine compared with the prone position at the SC segment (1.47 ± 0.07 m/s vs. 1.40 ± 0.09 m/s, *p* = 0.019). A 2 × 2 repeated‐measures ANOVA confirmed significant effects of both aortic segments (SC > IR, *p* < 0.05) and examination positions (supine > prone, *p* < 0.05) on SWS, with no significant interaction between the two factors (*p* = 0.090). Effect sizes with 95% confidence intervals and post hoc power estimates for all paired SWS comparisons are provided in Supplemental Table ST1.

The repeatability of MRE measurements was assessed in six volunteers who underwent two repeated scans at both the SC and IR segments in supine position, yielding 12 paired observations. Bland–Altman analysis demonstrated good agreement with a small, non‐significant bias (−0.031 m/s, *p* = 0.177) and narrow limits of agreement (−0.176 to 0.115) (Figure 5B). The intraclass correlation coefficient (ICC) further confirmed good reliability (ICC = 0.76, 95% CI 0.39–0.93), supporting consistent SWS quantification across repeated acquisitions.

Interobserver reproducibility of aortic wall segmentation was assessed in all subjects. SWS measurements derived from vessel wall segmentations performed independently by two observers demonstrated excellent agreement (ICC = 0.948, 95% CI: 0.869–0.975). Bland–Altman analysis revealed a negligible bias of 0.018 m/s with limits of agreement of −0.044 to 0.079 m/s (Figure 5C).

Based on the mixed‐effects model analysis, clinical variables listed in Table 1 showed no significant correlations with SWS in the group of young healthy volunteers. Age demonstrated a positive yet non‐significant trend (β ≈ 0.008 m/s per year, *p* = 0.10). In exploratory condition‐specific analyses, pulse pressure was inversely correlated with SWS in the supine SC condition (β ≈ −0.007 m/s per mmHg, *p* = 0.028), whereas no associations were observed for other conditions.

#### 3.2.2 Hemodynamic Characterizations of the Abdominal Aorta at Different Anatomical Segments and Examination Positions

**FIGURE 6:**
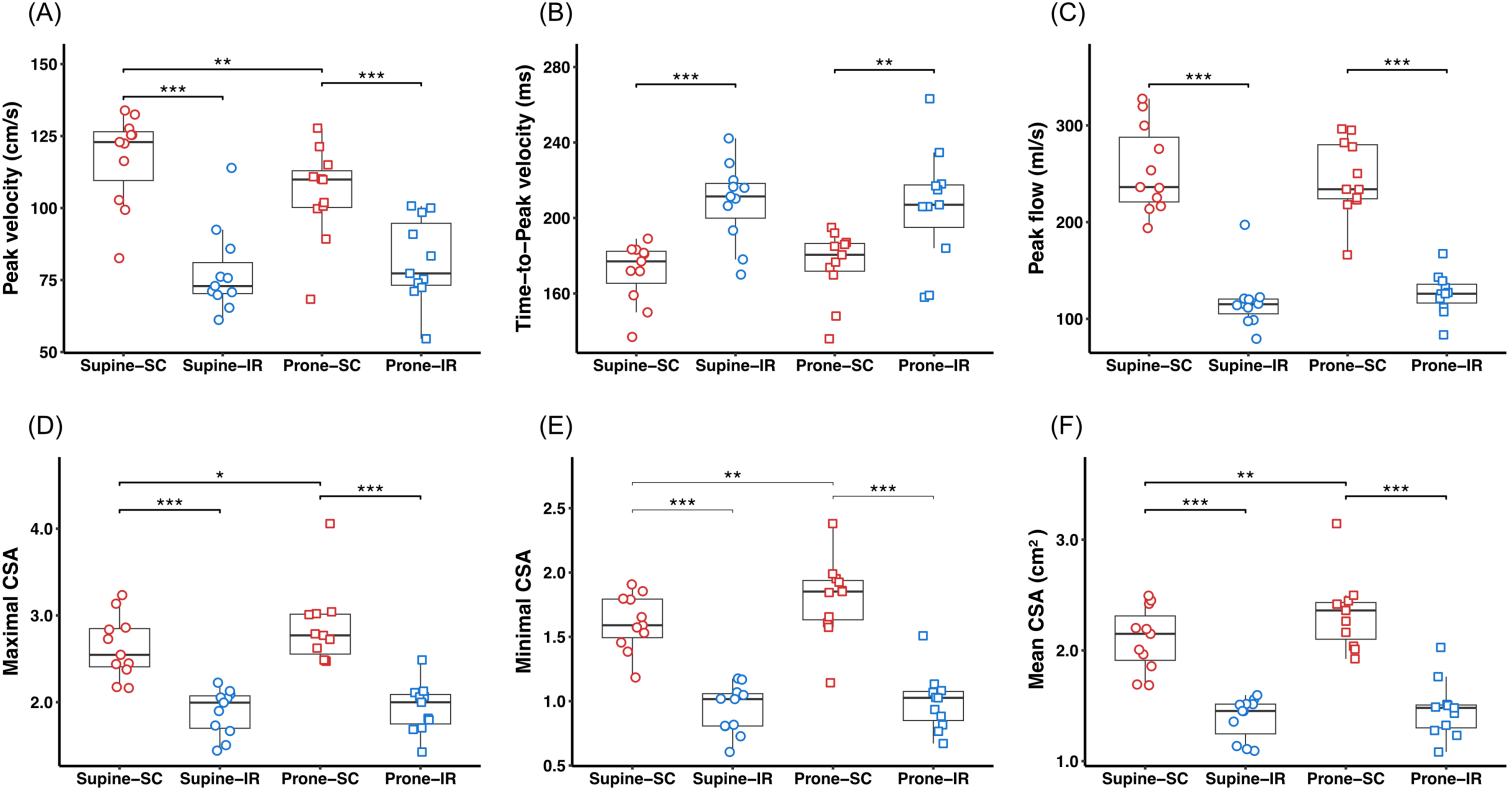
Hemodynamic parameters derived from flow‐encoded MRI at the supraceliac (SC, in red) and infrarenal (IR, in blue) aortic segments in supine (open circle) and prone (open square) positions. Boxplots show the group distribution of (A) peak velocity, (B) time‐to‐peak velocity, (C) peak flow, (D) maximal cross‐sectional area (CSA), (E) minimal CSA, (F) mean CSA. Significant differences between groups are indicated (*p < 0.05, **p < 0.01, ***p < 0.001).

Flow‐quantification imaging provided dynamic flow curves over the cardiac cycle that allowed quantification of velocity and vessel CSA parameters, shown representatively in Figure 3 for the same subject presented in Figure 4. The group averaged peak velocity and peak flow were significantly higher in the SC than in the IR segments, with a significant interaction between examination position and segment (*p* < 0.001) found in the peak velocity only. Based on condition specific pair‐wise comparison, peak velocity at the SC segment was higher in the supine than in the prone position (*p* < 0.001) (Figure 6A). Time‐to‐peak velocity was significantly lower in the SC segment than in the IR segment (*p* < 0.001) (Figure 6B). Peak flow, maximal, minimal and mean CSA were significantly lower at the IR segment than the SC segment, regardless of the examination positions (all *p* < 0.001) (Figure 6C–E). Changing positions from supine to prone only affected the maximal, minimal, and mean CSA of the SC segment (*p* < 0.05, *p* < 0.01 and *p* < 0.01) (Figure 6D–F). Detailed statistics pertaining to the group analysis of both biomechanical and hemodynamic parameters are collected in Table 2.

**TABLE 2:** Repeated measures ANOVA comparing group differences of shear wave speed (SWS) and hemodynamic parameters of the abdominal aorta at supraceliac (SC) and infrarenal (IR) segments in supine and prone positions.

Parameter (unit)	Supine–SC (Mean ± SD)	Supine–IR (Mean ± SD)	Prone–SC (Mean ± SD)	Prone–IR (Mean ± SD)	Position (supine vs. prone)	Segment (SC vs. IR)	Interaction (position × segment)
SWS (m/s)	1.47 ± 0.07	1.39 ± 0.13	1.40 ± 0.09	1.33 ± 0.10	F (1, 10) = 6.74, ηp^2^ = 0.40, ***p* = 0.027**	F (1, 10) = 7.26, ηp^2^ = 0.42, ***p* = 0.023**	F (1, 10) = 0.043, ηp^2^ = 0.004, *p* = 0.841
Peak velocity (cm/s)	117.4 ± 15.9	77.8 ± 14.8	105.0 ± 16.2	81.7 ± 14.6	F (1, 10) = 2.66, ηp^2^ = 0.210, *p* = 0.134	F (1, 10) = 55.15, ηp^2^ = 0.846, ***p* < 0.001**	F (1, 10) = 21.64, ηp^2^ = 0.684, **p < 0.001**
Time‐to‐peak velocity (ms)	171.3 ± 16.2	208.5 ± 21.1	175.4 ± 18.3	206.2 ± 30.7	F (1, 10) = 0.027, ηp^2^ = 0.003, *p* = 0.873	F (1, 10) = 63.39, ηp^2^ = 0.864, ***p* < 0.001**	F (1, 10) = 0.45, ηp^2^ = 0.043, *p* = 0.518
Peak flow (ml/s)	254.3 ± 45.2	117.5 ± 29.4	245.7 ± 39.6	125.9 ± 21.5	F (1, 10) = 0.29, ηp^2^ = 0.028, *p* = 0.60	F (1, 10) = 139.7, ηp^2^ = 0.933, ***p* < 0.001**	F (1, 10) = 0.14, ηp^2^ = 0.014, *p* = 0.72
Maximal CSA (cm^2^)	2.6 ± 0.4	1.9 ± 0.3	2.9 ± 0.5	1.9 ± 0.3	F (1, 10) = 7.49, ηp^2^ = 0.43, ***p* = 0.021**	F (1, 10) = 79.14, ηp^2^ = 0.89, ***p* < 0.001**	F (1, 10) = 3.02, ηp^2^ = 0.23, *p* = 0.113
Minimal CSA (cm^2^)	1.6 ± 0.2	0.9 ± 0.2	1.8 ± 0.3	1.0 ± 0.2	F (1, 10) = 7.82, ηp^2^ = 0.44, ***p* = 0.019**	F (1, 10) = 65.09, ηp^2^ = 0.87, ***p* < 0.001**	F (1, 10) = 2.45, ηp^2^ = 0.20, *p* = 0.148
Mean CSA (cm^2^)	2.1 ± 0.3	1.4 ± 0.2	2.3 ± 0.3	1.5 ± 0.3	F (1, 10) = 10.55, ηp^2^ = 0.51, ***p* = 0.009**	F (1, 10) = 94.75, ηp^2^ = 0.90, ***p* < 0.001**	F (1, 10) = 3.55, ηp^2^ = 0.26, *p* = 0.089

*Note:* Reported statistics include the F value with degrees of freedom, the corresponding *p* value, and partial eta squared (ηp^2^). SWS = shear wave speed; CSA = cross‐sectional area. *p*‐Values in bold are statistically significant.

### 3.3 Correlation Between the Biomechanical and Hemodynamic Parameters of the Abdominal Aorta

**FIGURE 7:**
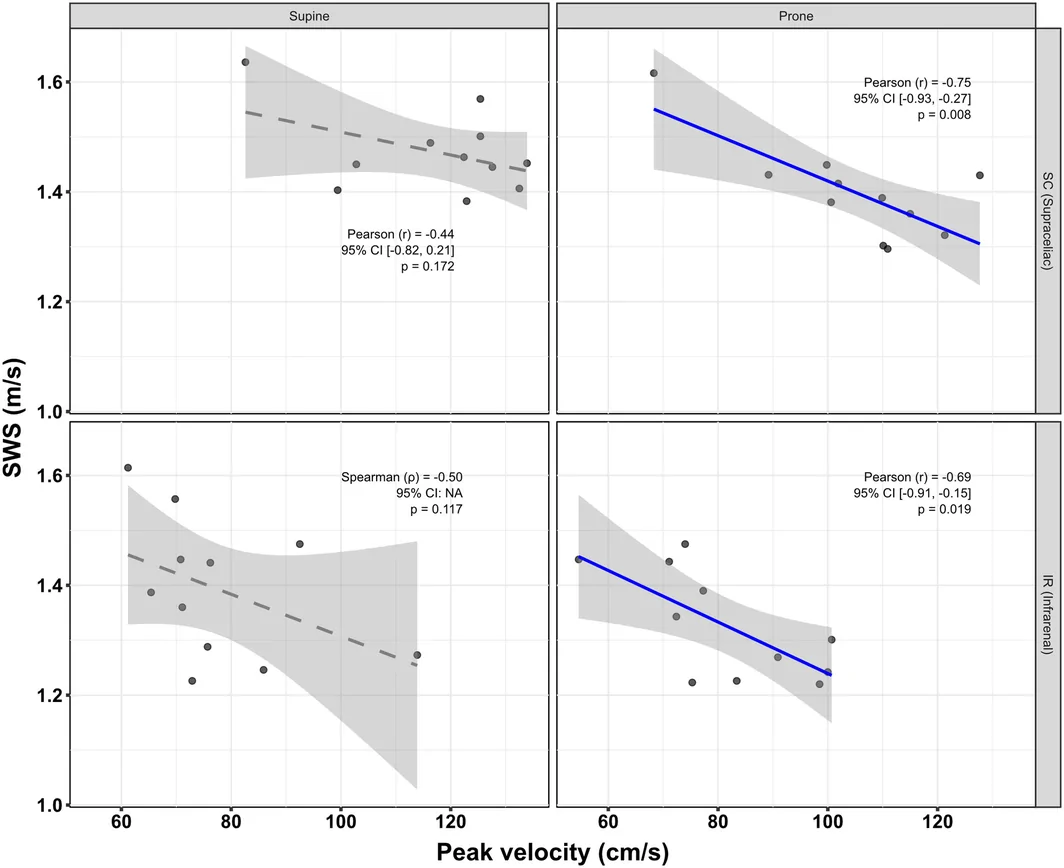
Correlation between shear wave speed (SWS) and peak velocity at supraceliac (SC) and infrarenal (IR) aortic segments in supine and prone positions. Linear regression fits are overlaid for visualization: solid blue lines indicate statistically significant associations (*p* < 0.05), and dashed gray lines indicate non‐significant associations (ns, *p* ≥ 0.05). Shaded areas represent 95% confidence bands of the fitted regression lines.

Repeated‐measures correlation analysis pooling all data together revealed a series of correlations between SWS and hemodynamic parameters including peak velocity (r = 0.37, 95% CI 0.04–0.63, *p* = 0.031), time‐to‐peak velocity (r = −0.34, *p* = 0.048), peak flow (r = 0.42, *p* = 0.014), maximal CSA (r = 0.39, *p* = 0.022), and mean CSA (r = 0.36, *p* = 0.039). After adjusting for examination position and anatomical segments, only peak velocity remained independently correlated with SWS (*p* = 0.001). In subgroup analyses with respect to examination positions, negative correlations between SWS and peak velocity were observed in the prone position at both SC and IR segments (SC: r = −0.75, 95% CI –0.93 to −0.27, *p* = 0.008; IR: r = −0.69, 95% CI –0.91 to −0.15, *p* = 0.019), whereas no significant correlations were found in the supine position (Figure 7). Results pertaining to the linear mixed‐effects model analysis and the subgroup correlation analysis are summarized in Table 3.

**TABLE 3:** Correlations between shear wave speed (SWS) and hemodynamic parameters.

Parameter (unit)	Repeated‐measures correlation r (95% CI)	*p*	LME model (adjusted for posture + segment)	Within‐condition correlations (*n* = 11)
Peak Velocity (cm/s)	r = 0.37 (0.04, 0.63)	0.031	β = −0.071, ***p* = 0.001**	Prone‐SC: r = −0.75, ***p* = 0.008 (FDR = 0.033)**; Prone‐IR: r = −0.69, ***p* = 0.019 (FDR = 0.037)**; others ns
Time‐to‐peak Velocity (ms)	r = −0.34 (−0.61, −0.00)	0.048	β = −0.010, *p* = 0.626 (ns)	Prone‐SC: r = −0.73, ***p* = 0.010 (FDR = 0.041);** others ns
Peak Flow (ml/s)	r = 0.42 (0.09, 0.66)	0.014	β = −0.050, *p* = 0.186 (ns)	ns
Maximal CSA (cm^2^)	r = 0.39 (0.06, 0.64)	0.022	β = 0.042, *p* = 0.114 (ns)	Prone‐IR: r = 0.71, *p* = 0.014 (FDR = 0.055); others ns
Minimal CSA (cm^2^)	r = 0.34 (0.00, 0.61)	0.052	β = 0.009, *p* = 0.745 (ns)	ns
Mean CSA (cm^2^)	r = 0.36 (0.02, 0.62)	0.039	β = 0.036, *p* = 0.212 (ns)	ns

*Note:* Repeated‐measures correlation (rmcorr) was used to assess within‐subject associations across the four measurement conditions. Linear mixed‐effects models (LME) were applied to adjust for posture (supine vs. prone) and anatomical segment (supraceliac vs. infrarenal). Within‐condition Pearson's correlations are reported separately for each subgroup (*n* = 11), and *p*‐values were corrected using false discovery rate (FDR). *p*‐Values and FDR in bold are statistically significant, ns: not significant.

## 4 Discussion

This study demonstrated the feasibility of abdominal aortic wall MRE in healthy volunteers and provided new insights into the influence of anatomical segments and examination positions on aortic wall stiffness, as well as the relationship between wall stiffness and flow‐derived hemodynamic parameters. We optimized the MRE sequence by implementing several technical modifications to enable more accurate and robust characterization of the abdominal aortic wall. The main findings were as follows: (1) aortic wall MRE with blood suppression was feasible and showed very good repeatability; (2) the supraceliac (SC) segment of the aortic wall was stiffer than at infrarenal (IR) segment position; (3) measurements performed in the supine position yielded higher wall stiffness than those conducted in the prone position, interestingly only at the SC segment; (4) in the mixed‐effects model, peak velocity was identified as an independent factor that negatively correlated with aortic wall stiffness.

MRE is a phase‐contrast–based sequence that encodes the tissue displacements induced by defined mechanical driving frequencies. In the aorta, pulsating blood flow might influence the encoded displacement. To minimize the influence of blood, second‐order moment‐nulling motion encoding gradients were employed, and the MRE acquisition windows were timed to the mid‐to‐late diastole, when the blood velocity was low, and the vessel wall exhibited minimal motion. With this acquisition strategy, the stiffness values obtained for aortic wall were well repeatable in our cohort. Our strategy enabled efficient acquisition of wave propagation and robust reconstruction of stiffness maps, with minimal influence from BMI. In our cohort, a volunteer with a high BMI of 29.1 kg/m^2^ also exhibited clearly discernible tissue‐wall structures on the stiffness maps, with stiffness values comparable to those of the other participants (Supplemental Figure SF3). Compared with previous publications [12, 18, 19], the aortic wall stiffness reported in the current study was slightly lower. This difference may be attributed to the differences in acquisition strategy and the aortic wall coverage. In previous studies [12, 18, 19], the stiffness of the aorta was assessed by combining the signals from both the aortic wall and the lumen as one effect media. This method is based on the waveguide effect, which assumes that the aortic wall and the blood inside the lumen vibrate in synchrony. As seen in the stiffness maps presented in [18, 19], the high stiffness signals are predominantly located within the lumen, whereas the stiffness within the aortic wall is comparatively lower. In our MRE acquisition, the blood signal in the lumen was nulled out and our region of interest was exclusively placed on the aortic wall. Furthermore, earlier research has characterized the aorta in the sagittal plane, covering a more extensive section of the vessel. In our study, the aorta was measured in a 2‐mm axial slice to effectively suppress the blood signal with a single trigger delay time. Since different aortic segments may exhibit distinct stiffness due to variations in wall structure and flow‐related pulsatile loading, as evidenced by the present study, our lower value may be due to differences in aortic coverage.

Our data revealed significant regional differences in abdominal aorta stiffness. This difference in apparent wall stiffness could reflect structural and physiological variations along the abdominal aorta. The SC segment is exposed to higher pulsatile loading and is more perivascularly constrained, features that enhance load‐bearing capacity and wall strength. Our observation is consistent with prior ex vivo experimental work in mice [28]. Furthermore, unlike ex vivo mechanical testing where the abdominal aorta was investigated under load‐free and stress‐free configurations, in vivo MRE quantified apparent aortic wall stiffness also reflects the wall's loading imposed by blood flow. Despite the implementation of a delay time during the RR interval to ensure low flow velocity for the MRE acquisition, the blood flow remains comparatively higher in the SC segment compared to the IR segment (Supplemental Figure SF4). This elevated flow can lead to increased loading, which may contribute to the observed apparent high stiffness in SC segment. The apparent in vivo wall stiffness obtained in the current study could reflect biomechanical wall strength and integrity, which may be associated with segmental susceptibility of AAA. In the presence of age‐related extracellular matrix degradation and adverse hemodynamic stresses [25], apparent in vivo wall stiffness may contribute to the clinical observation that AAA most commonly arise in the infrarenal aorta [3].

Regarding the examination positions, lower aortic wall stiffness was observed in the prone position compared to the supine position in the SC segment. Furthermore, significant correlations between aortic wall stiffness and hemodynamic parameters were only identified in the prone position. Instead of exerting direct compression on the abdominal aorta, the prone posture has been shown to change central hemodynamics, reducing wave reflection and pulsatile afterload [29]. Elevated intra‐abdominal pressure may also lead to changes in venous return and cardiac preload. We therefore speculate that these hemodynamic changes, and the resulting reduction in pulsatile loading, may have contributed to the observed decrease in apparent in vivo stiffness. However, the relationship between MRE‐derived stiffness and hemodynamic variability remains to be established and will require further investigation through dedicated phantom studies and carefully controlled in vivo experiments, which are beyond the scope of the present study. Previous studies have demonstrated that body posture substantially impacts abdominal vascular hemodynamics. Kadoya et al. reported that the upright position significantly reduced abdominal aortic mean and peak velocities in healthy volunteers [30]. A recent review further emphasized the influence of posture on vascular geometry and hemodynamics [31]. Nevertheless, direct comparisons between prone and supine examination positions remain very limited. The present study offers the first evaluation of posture‐related variations in abdominal aortic wall stiffness and blood flow in healthy adults.

Additionally, aortic wall stiffness exhibited a negative correlation with peak velocity. This finding could suggest that increased wall stiffness reduces vascular distensibility, thereby limiting systolic expansion and consequently diminishing flow acceleration. This effect is also evident in the flow waveform which became less peaked, resulting in a lower peak velocity. This same phenomenon has also been observed in elderly healthy individuals with increased aortic stiffness, particularly in the descending aorta [32]. It is important to note that pulse wave velocity, another independent hemodynamic parameter that increases with aging and high wall stiffness, is not necessarily correlated with the peak velocity reported in the current study [33].

The present study has limitations. First and foremost, this technical feasibility study included a relatively small cohort consisting exclusively of healthy young male volunteers, which limits the generalizability of the findings and precludes assessment of the proposed methodology in pathological conditions. Subsequent studies are planned to involve patients with abdominal aortic aneurysm, atherosclerosis, or inflammatory disease. Secondly, the trigger delay was selected based on a series of scout images, which renders our imaging protocol complex. Nevertheless, according to our findings, it is conceivable that the user could reduce the number of scout images based on the appearance of the flow–time curves from the cine acquisition, which is in the standard cardiovascular imaging workflow. Thirdly, images of the IR segment were more susceptible to motion artifacts and B_0_ inhomogeneity than that of the SC segment due to bowel peristalsis and intestinal filling status. Bowel preparation prior to the examination could be implemented as a viable solution in a clinical setting if necessary. Fourth, even with careful ROI placement, the limited spatial resolution of MRE prevents complete elimination of partial volume effects. However, this limitation is expected to be less pronounced in conditions with increased aortic wall thickness, such as atherosclerosis and hypertension.

In summary, this study demonstrated the feasibility and good repeatability of abdominal aortic wall MRE in healthy volunteers, providing wall stiffness with a minimal contribution from blood. The findings of this study demonstrated the dependence of aortic wall stiffness on distinct anatomical segments and examination positions. These results underscored the potential of imaging‐based biomechanical parameters to further our understanding of vascular health. However, the clinical utility and advantages of this approach over existing methods remain to be established in patient studies. Further validation in larger cohorts, including patients with vascular disease, is warranted to determine the value of aortic wall MRE for risk stratification, disease monitoring, and clinical decision‐making.

## Funding

This work was supported by Deutsche Forschungsgemeinschaft (10.13039/501100001659; GRK2260 BIOQIC), SFB1340 Matrix‐in‐Vision (FOR5628), and Heisenberg Grant (GU 1726/5‐1; 504873209).

## Conflicts of Interest

Ingolf Sack and Jing Guo are cofounders of THEA‐Devices GmbH, Germany. Jens Vogel‐Claussen receives research funding from the NIH, BMBF (DZL), AstraZeneca, GSK, Boehringer Ingelheim, honoraria from Siemens Healthineers, AstraZeneca, GSK, Roche, Coreline Soft, IQWIG, Deutsche Röntgengesellschaft, Bayer. Jens Vogel‐Claussen is the cofounder of BioVisioneers GmbH. All the other authors have no conflicts of interest to declare.

## Supporting information


**Figure SF1:** MRE displacement at all driving frequencies and three encoding directions, overlaid on the magnitude images.
**Figure SF2:** Animated shear wave propagation at all driving frequencies within the aortic wall of the supraceliac and infrarenal segments in the same subject shown in Figure 4.
**Figure SF3:** Representative shear wave speed (SWS) maps of the aorta from a healthy control volunteer with a relatively high body mass index (BMI = 29.1). The left panel displays the SWS map at the supraceliac segment (1.5 m/s), whereas the right panel shows the infrarenal segment (1.3 m/s).
**Figure SF4:**: Flow velocity during MRE acquisition for all volunteers at the supraceliac (SC, in red) and infrarenal (IR, in blue) aortic segments in supine (open circle) and prone (open square) positions. Significant differences between groups are indicated (*p < 0.05, **p < 0.01, ***p < 0.001).
**Table ST1:** Post hoc power analysis for paired comparisons of shear wave speed (SWS) between aortic segments and body positions.

## Data Availability

The data that support the findings of this study are available from the corresponding author upon reasonable request.
