# Supplementary material for: Multifrequency Magnetic Resonance Elastography of the Abdominal Aortic Wall: Segmental, Positional and Flow Effects
Source: NMR Biomed. 2026 Sep 17;39(10):e70401. doi: 10.1002/nbm.70401 (PMC13584248; doi:10.1002/nbm.70401)
Supplement: Supplementary file 1 [file NBM-39-e70401-s001.docx]

**Supplementary Material**

**Supplemental Figure SF1**. MRE displacement at all driving frequencies and three encoding directions, overlaid on the magnitude images.

**Supplemental Figure SF2:** Animated shear wave propagation at all driving frequencies within the aortic wall of the supraceliac and infrarenal segments in the same subject shown in Figure 4.

**Supplemental Figure SF3:** Representative shear wave speed (SWS) maps of the aorta from a healthy control volunteer with a relatively high body mass index (BMI = 29.1). The left panel displays the SWS map at the supraceliac segment (1.5 m/s), while the right panel shows the infrarenal segment (1.3 m/s).

**Supplemental Figure SF4**. Flow velocity during MRE acquisition for all volunteers at the supraceliac (SC, in red) and infrarenal (IR, in blue) aortic segments in supine (open circle) and prone (open square) positions. Significant differences between groups are indicated (*p < 0.05, **p < 0.01, ***p < 0.001).

**Supplemental Table ST1:** Post-hoc power analysis for paired comparisons of shear wave speed (SWS) between aortic segments and body positions.

| Comparison | Test | N | P | Effect type | Effect size | Effect CI | Post-hoc Power |
| --- | --- | --- | --- | --- | --- | --- | --- |
| Supine Supraceliac vs Supine Infrarenal | Paired-T | 11 | 0.035 | Cohen's dz | 0.735 | [0.050, 1.392] | 0.595 |
| Prone Supraceliac vs Prone Infrarenal | Paired-T | 11 | 0.0462 | Cohen's dz | 0.686 | [0.011, 1.334] | 0.538 |
| Supine Supraceliac vs Prone Supraceliac | Paired-T | 11 | 0.0192 | Cohen's dz | 0.84 | [0.132, 1.519] | 0.71 |
| Supine Infrarenal vs Prone Infrarenal | Paired-T | 11 | 0.0899 | Cohen's dz | 0.566 | [-0.086, 1.194] | 0.397 |
